# Species composition and vegetation structure of coastal and desert habitats in a hyper-arid environment

**DOI:** 10.1038/s41598-026-36782-x

**Published:** 2026-03-09

**Authors:** Abdelraouf A. Moustafa, Samira R. Mansour, Monier M. Abd El-Ghani

**Affiliations:** 1 Department of Botany and Microbiology, Faculty of Science, Suez Canal University, Ismailia, Egypt; 2 Department of Botany and Microbiology, Faculty of Science, Cairo University, Giza, Egypt

**Keywords:** Floristic diversity, Mediterranean coast, Inland desert, Habitat diversity, Vegetation analysis, Egypt, Ecology, Ecology, Environmental sciences

## Abstract

Environmental changes and anthropogenic activities have significantly altered plant species distributions and biodiversity in various regions of Egypt, contributing to habitat fragmentation and a decline in plant populations. This study provides a comprehensive assessment of vegetation diversity, species distribution, conservation status, and environmental relationships across diverse habitats in Egypt, including coastal regions (Hurghada and El-Arish) and inland deserts (Wadi El-Gemal and El-Galala). From 2023 to 2024, a vegetation survey was conducted across four study areas in Egypt using stratified random sampling; the survey involved 86 sampled plots, analyzing species composition, growth forms, and cover. A total of 45 species from 16 families were recorded, with Asteraceae, Amaranthaceae, and Zygophyllaceae being the most dominant. Species were classified as native or non-native, and their conservation status was determined based on the IUCN Red List criteria. Multivariate analyses (cluster analysis, Detrended Correspondence Analysis, and Canonical Correspondence Analysis) revealed seven distinct vegetation groups (A–G), which were significantly influenced by soil properties such as pH, electrical conductivity, and ion content. The study also calculated diversity indices, with average species richness (SR) of 4.5 ± 2.1 species per plot and a Shannon–Wiener Index (H’) ranging from 0.38 to 1.99. Conservation status assessments showed that 95.5% of species were native, with two non-native species recorded. Several species, such as *Anabasis articulata* and *Haloxylon salicornicum*, were categorized as Vulnerable (VU), while others, including *Panicum turgidum* and *Phragmites australis*, were classified as Near Threatened (NT) or Least Concern (LC). The results of this study underscore the importance of understanding the complex relationships between plant communities and environmental factors to develop targeted conservation strategies. The findings also emphasize the need for continued monitoring and protection of both coastal and inland desert habitats in Egypt to mitigate the effects of environmental degradation and preserve biodiversity.

## Introduction

Egypt, despite its hot and arid climate, is home to about 2121 plant species from 758 genera^1^. Geographically, Egypt occupies a strategic location at the crossroads of Africa, Asia, and Europe, situated in the northeastern part of Africa. This positioning facilitates connections between the Mediterranean and sub-Saharan Africa through the Nile Valley and links the tropical Indian Ocean to the Mediterranean via the Red Sea. Additionally, Egypt borders the Mediterranean Sea, the Red Sea, and the Gulfs of Suez and Aqaba, further enhancing its ecological and geopolitical significance^2^. The country’s diverse desert plains and mountain systems harbor a wide range of ecosystems and habitat types. Major habitats include arid desert landscapes, rugged mountainous regions, plains, slopes, sand dunes, and salt marshes^2^. Aquatic ecosystems are represented by the Nile River and its extensive network of tributaries, canals, and drains, as well as freshwater and brackish lakes, wetlands, and the Mediterranean and Red Sea coasts^4^. Urban habitats, particularly those in the densely populated Nile Valley, such as canal and drain banks, roadsides, and railway corridors, also contribute to Egypt’s biodiversity^2^. This ecological variety underscores Egypt’s role as a biodiversity hotspot, supporting numerous plant and animal species adapted to its unique environmental and climatic conditions.

Environmental changes and anthropogenic activities are major drivers of biodiversity loss, impacting plant species across various ecosystems^5^. In Egypt, these factors have led to habitat fragmentation and a significant decline in the number of plant species, particularly in regions characterized by fragile ecosystems such as coastal areas and inland deserts^6^. The Mediterranean and Red Sea coasts, including the El-Arish and Hurghada regions, as well as inland deserts like Wadi Al Galala and Wadi El-Gemal, are particularly vulnerable to these changes. These ecosystems are under pressure from both climate change and human activities, which alter vegetation composition, disrupt species distribution, and threaten the survival of native plant species^2^.

The vegetation of the Red Sea and Eastern Desert has been the focus of numerous Egyptian researchers due to its ecological significance and unique adaptations^7^^,^^8^. The Red Sea, compared to many other tropical and subtropical seas, boasts a rich and diverse environment. Zahran et al.^9^ categorize the coastal lands of the Red Sea into two primary habitat types: saline and non-saline. These habitats exhibit exceptional floristic richness and biodiversity. The Red Sea’s elevated salinity, a result of its arid climate and limited freshwater inflow, supports the proliferation of halophytic species, such as saltbush and seagrass. This hyper-salinity also creates specialized conditions that favor unique marine organisms and coastal vegetation^10^.

Despite its ecological importance, the rugged topography and inaccessibility of the region’s rocky escarpments have limited floristic studies, particularly addressing altitudinal vegetation diversity. The distribution, abundance, and community composition of plant species in these desert ecosystems are strongly influenced by both abiotic factors, such as soil salinity, water availability, altitude, and anthropogenic activities^11^^,^^12^. Human-induced disturbances, including overgrazing and unsustainable plant harvesting, have significantly altered the natural vegetation cover over time. Salt marsh vegetation along the Red Sea is dominated by species like *Zygophyllum album* and *Nitraria retusa*. *Zygophyllum album*, a salt-tolerant species, plays a critical role in sand dune stabilization and is confined to salt marsh habitats. *Nitraria retusa,* a halophytic succulent shrub, often forms large sand hillocks, surpassing the size of the mounds created by *Zygophyllum album*. In the coastal desert plains, vegetation thrives primarily in runnels where runoff water accumulates, within an altitudinal range of 10–300 m above sea level (a.s.l.). A notable species in this habitat is *Cornulaca monacantha*, which dominates the vegetation in these low-lying areas. The intricate interplay of abiotic and biotic factors in this region underpins the complex and dynamic vegetation patterns, highlighting the need for further systematic studies to understand and conserve these unique ecosystems.

The Mediterranean region is recognized as one of the world’s 25 biodiversity hotspots 13. Its diverse coastal habitats support between 4 and 18% of the planet’s biodiversity, making it a global center for ecological richness. The region harbors over 25,000 vascular plant species, approximately 5,500 of which are endemic, ranking it as the third most significant biodiversity hotspot for plant diversity worldwide. Despite this extraordinary diversity, significant gaps remain in understanding the vascular plant diversity of the Mediterranean basin. Spatial and temporal disparities in botanical exploration across the vast and heterogeneous Mediterranean territories emphasize the need for localized research to investigate, monitor, and conserve its plant diversity^13^. According to^14^ UNEP (2022), the Mediterranean is one of the world’s most sensitive regions to climate change. The combination of significant human activity and the deep historical interplay between the region’s landscapes and flora has shaped its biodiversity over millennia. However, contemporary challenges such as population expansion and rapid economic development are driving unprecedented anthropogenic changes. These factors are altering biodiversity patterns and disrupting plant population distributions. Vegetation along the Mediterranean coastline typically *comprises maquis*, a dense shrubland, and garrigue, a more open, scrubby vegetation type. Both vegetation types are increasingly under threat from climate and human pressures.

In Egypt, the impacts of climate change and human activities pose serious threats to biodiversity, particularly to plant communities. To accurately characterize the bioclimatic conditions of the studied regions, climate data were derived from long-term climatological records. Following standard climatological practices, 30-year averages were used to represent baseline climatic conditions, allowing for a robust assessment of temperature and precipitation patterns and minimizing the influence of short-term variability. Such long-term climate normals provide a reliable framework for evaluating the impacts of climate change on vegetation distribution and ecosystem dynamics. According to the Köppen–Geiger climate classification, the study areas in Egypt fall within two main climatic zones. Hurghada, Wadi El-Gemal, and El-Galala are classified as hot desert climate (BWh), characterized by extremely low precipitation, high evapotranspiration, and persistently high temperatures, which favor xerophytic and halophytic vegetation. In contrast, El-Arish, located along the Mediterranean coast, belongs to the Mediterranean hot-summer climate (Csa), with relatively higher winter rainfall and milder temperatures. This climatic contrast provides an appropriate framework for assessing vegetation heterogeneity and plant distribution patterns across coastal and inland desert ecosystems. Climate change affects specific species, their ecological interactions, habitats, and overall ecosystem functioning, reducing the capacity of natural systems to provide essential goods and services for society^15^. Anthropogenic influences, including habitat fragmentation, mining, poaching, unsustainable grazing and harvesting, urbanization, pollution, infrastructure development, and illegal activities such as vandalism and squatting, further exacerbate these pressures. Activities such as trampling, fishing, and disturbance from construction and emergencies have led to the depletion of natural resources, environmental degradation, and significant genetic erosion^16,17^. These combined pressures have resulted in the extinction of numerous pastoral plant species and a marked scarcity of trees and shrubs, underscoring the urgent need for effective biodiversity conservation measures. Establishing and managing natural protectorates is critical to preserving biodiversity. Effective management plans (MPs) must incorporate comprehensive ecological assessments and data analysis to evaluate the status of protected areas and implement adaptive strategies to mitigate threats^16^. By addressing these challenges, Egypt can safeguard its natural heritage and ensure the resilience of its ecosystems in the face of ongoing environmental and anthropogenic pressures^18^. Understanding the status of plant biodiversity in these regions is crucial for developing effective conservation strategies. This study aims to provide a comprehensive assessment of vegetation heterogeneity, species distribution patterns, conservation status, nativity of species, and plant species diversity in different habitats in Egypt, at coastal regions as: (Hurghada and El-Arish,) and inland regions: (Wadi El-Gemal and El Galala) desert ecosystems in Egypt using multivariate analysis techniques.

### Study areas

#### El-Arish (coastal desert)

**Fig. 1:**
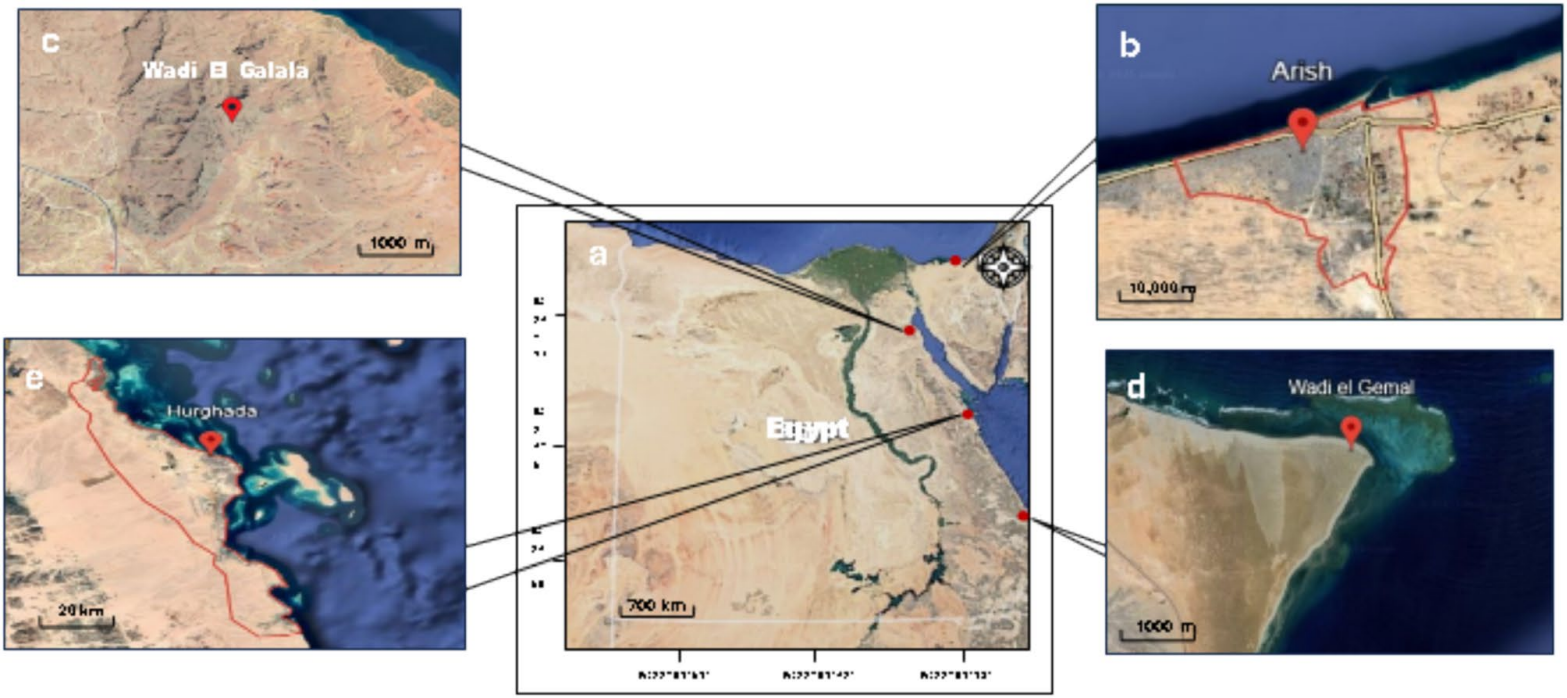
**A** Satellite map of Egypt, **B** El-Arish, the coastal region that is situated along the Mediterranean Sea, **C** Galala Mountain, the inland desert located in the eastern desert, **D** Hurghada, Costal area positioned along the Red Sea coast, **E** Wadi El-Gemal National Park (WGNP), which located in the Eastern Desert. *Source*: https://www.google.com/earth/

El-Arish is the capital and largest city of the North Sinai Governorate of Egypt (Fig. 1), as well as the largest city on the Sinai Peninsula, lying on the Mediterranean coast 344 km northeast of Cairo and 45 km west of the Egypt–Gaza border. Although Al-Arish is a desert, some of it has recently been restored, while others are cultivated. It is located between longitude 33° 40’ and 34^o^E, and latitude 31° and 31° 9’ N^19^. El-Arish maintains its classification as a hot desert climate, specifically BWh according to the Köppen climate classification system. The site claims to be on the northeastern border of Egypt. As anticipated, considering its geographical location and classification, the climate data for the recent standard 30-year normal period Average Annual Temperature: approximately 19.5 °C. Temperature Range: Average monthly temperatures vary by about 13.6 °C, warmest month (August): average high around 31–32 °C. Coolest Month (January): average high around 19 °C and low around 9 °C, annual Precipitation: averages around 106 mm to 147 mm (4.2 to 5.8 inches) per year. Rainfall is minimal and highly seasonal, with the majority occurring in the winter months (e.g., January averages about 33 mm), Summer months (June, July, August) typically see negligible to zero precipitation. The classification remains consistent with the data you provided, reinforcing that El-Arish is defined by extremely low rainfall and high temperatures, meeting the criteria for a BWh climate type^20^.

**Fig. 2:**
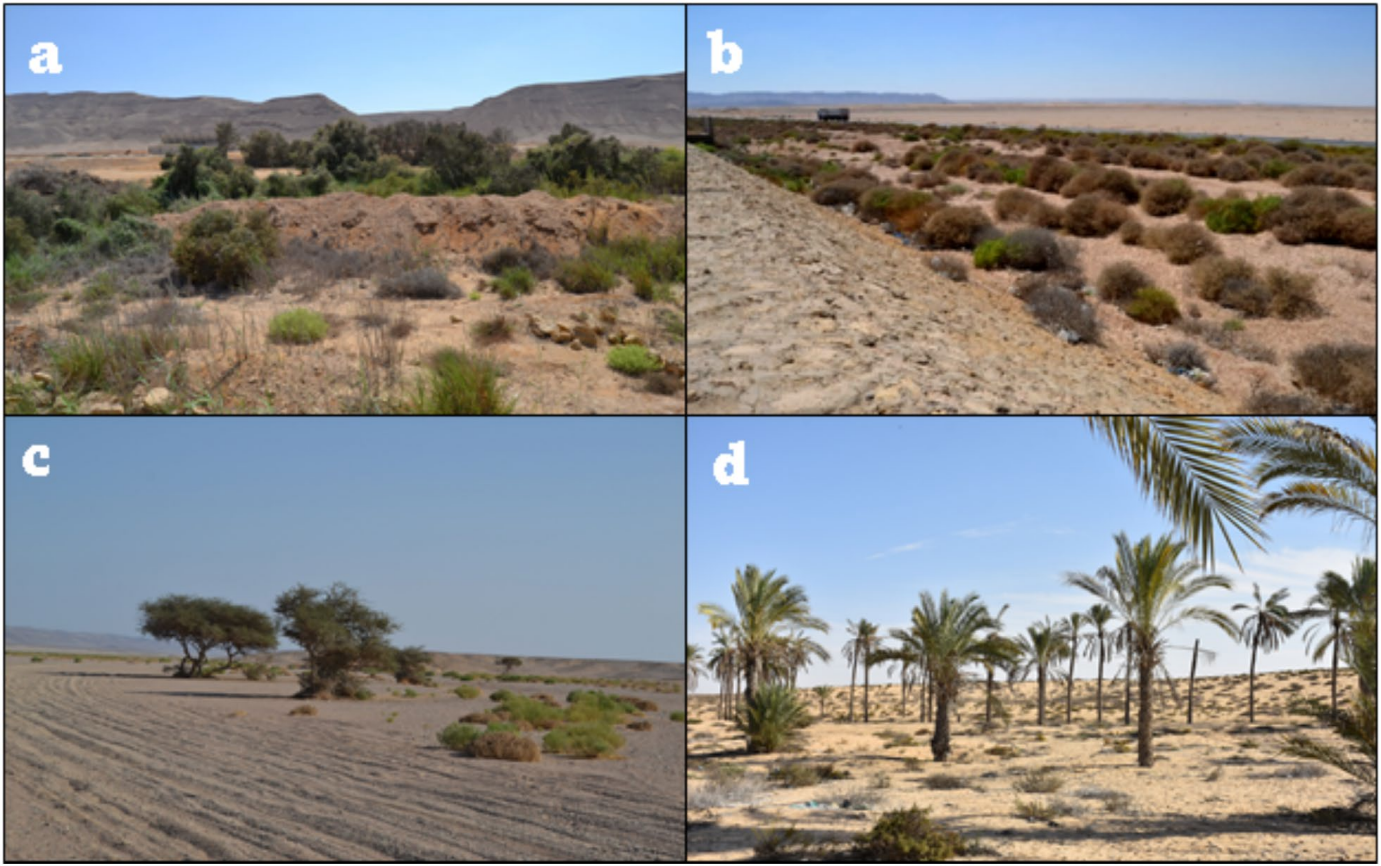
The habitats of the 4 locations of the Study area. **A** Hurghada (coastal desert), **B** Wadi El-Gemal (inland desert), **C** Wadi El Galala (inland desert), **D** El-Arish (coastal desert). *Source* These photos were taken by Abdelraouf Moustafa.

The El-Arish coastal region has unique geomorphology influenced by geological processes, climatic influences, and human activity. Its main coastline consists of long sandy beaches, coastal dunes, lagoons, and wetlands, including the El-Arish lagoon, which is formed by sediment and freshwater accumulation from rivers and rain (Fig. 2). Some areas along the coastline have rocky outcrops, possibly limestone or sandstone formations, contrasting with the sandy beaches. The costal dunes are characterized by sandy soils, strong winds, and saltwater exposure and include some species such as *Panicum turgidum*, *Nitraria retusa* and *Tamarix nilotica*.

#### Wadi El Galala (inland desert)

Al Galala Mountain is located in the Red Sea Governorate of Egypt (Fig. 1), about 80 km south of Suez, with an elevation that reaches about a height of 1,470 m (4,823 feet) above sea level. It is composed mainly of sandstone and limestone (Fig. 2). It features rugged terrain with deep canyons and valleys. Wadi El Galala is a wadi (valley) that runs through El Galala Mountain, approximately 15 km long. It’s a narrow gorge with steep cliffs and a dry riverbed and supports a variety of plant and animal life, including *acacia* trees, desert foxes, and ibex^22^. It considered the home to various animal and plant species adapted to the harsh arid conditions, including some endemic ones and had sources of water that have since dried up. It includes (*Acacia tortilis*, *Zygophyllum coccineum*, *Tamarix nilotica*, *Nitraria retusa*, *Artemisia herba-alba*, *Anabasis articulata* and *Astragalus sinaicus)* and many other species. Annual plants are vital in this arid habitat because of their quick developmental cycles and effective resource utilization. Their rapid development and growth enable effective water absorption, resulting in less water loss through runoff^22^. The studies were carried out on the narrow gorge through the Galala Mountain, and the surrounding wadies include Wadi El Galala. Wadi El Galala has a hot desert climate (BWh) according to the Köppen climate classification system. Climate data for the past 30 years (approximately 1995–2025) confirm extremely low precipitation: Rainfall is very scant, often only a few tens of millimeters per year, or virtually zero. Very high temperatures: The region experiences high temperatures, especially during summer months (May to September), with average annual temperatures generally above 18 °C. Daytime highs can be very hot. High isolation: low humidity: Relative humidity is generally very low in desert regions^20^.

#### Hurghada (coastal desert)

Hurghada serves as a city and attraction for visitors on Egypt’s Red Sea coastline (Fig. 1). It was founded in the early twentieth century, and since the 1980s, it has transformed from a desert area and basic fishing village to an expanding modern city. Hurghada International Airport serves the city, which is the administrative head office of the Red Sea Governorate (RSG). The Hurghada area is primarily made up of three administrative sectors: El-Gouna resort, Hurghada city, and Sahl Hashish area, which has expanded 60 km down the shoreline^23^. Climatologically: Hurghada, Egypt, has a hot desert climate, which is classified as BWh under the Köppen climate classification system. Climate data for the past 30 years confirm Arid Conditions: The region receives extremely low annual precipitation, typically less than 50 mm, with some estimates as low as 6 mm per year. Rainfall is minimal and highly irregular, High Temperatures: Summers (May to September) are long, hot to very hot, with average high temperatures often exceeding 35 °C, peaking in August. Winters are mild and warm, with daily maximum temperatures around 20–25 °C , large Diurnal Range: There is a significant difference between daytime and nighttime temperatures, especially in winter months, where evening temperatures can drop considerably^21^. The region is characterized by a desert climate with limited rainfall and salty soil, which significantly influences plant life such as *Tamarix nilotica*, *Zygophyllum coccineum*, *Halophila ovalis*, and many other plants. The Hurghada region is divided into three geomorphological divisions: coastal plain, pediment, and hilly terrain. The coastal plain is wide and covered in Pleistocene reefal limestone, gravel, sands, and recent deposits. The pediment is an inclined plain with potential for urban expansion. The mountainous terrain is made up of Precambrian basement rocks and features the second-highest peak in Egypt, Gabal Shayeb al-Banat^23^ (Fig. 2).

#### Wadi El-Gemal (inland desert)

Wadi El-Gemal National Park (WGNP) was established in 2003.WGNP covers 4,770 km^2^ of land and about 2,000 km2 of marine waterways. The park’s ranger station, main gate, and visitor center are all located approximately 50 km south of Marsa Alam, Egypt (Fig. 1). WGNP is between latitudes N 24° 5’ and N 24° 53’. It is also part of the hyper-arid region, which has an arid climate with scorching, rainless summers and mild winters, and is home to diverse biodiversity, culture, and traditions. The geomorphology of Wadi El-Gemal has had a significant impact on the region’s biodiversity and ecology. The diverse geological features have created a variety of habitats, supporting a wide range of plant and animal species. The Wadi El-Gemal is a narrow valley formed by sandstone and limestone erosion, surrounded by rugged mountains, including the highest point in Egypt, the Gebel Elba range. The region features numerous canyons and gorges, sand dunes, and steep cliffs along the Red Sea coast, shaped by prevailing winds and vegetation^24^ (Fig. 2). Wadi El-Gemal falls under the hot desert climate classification, designated as BWh by the Köppen system. Climate Characteristics (Last 30 Years), Overall Conditions: The area is characterized by a hyper-arid climate with extremely low precipitation, high temperatures, and high evaporation rates. Precipitation: Rainfall is very rare and often occurs as heavy, short-duration showers, primarily in the autumn and winter months, which can lead to flash floods. The annual rainfall quantity is minimal (around 17.4 mm). Temperature: The region experiences hot, rainless summers and mild winters, Mean annual temperature (MAT) is typically above 18 °C, which is the criterion for the “h” (hot) designation^20^.

Overall, the studied areas encompass contrasting desert environments in Egypt, differing markedly in climatic conditions, geomorphology, and vegetation structure. El-Arish, located along the Mediterranean coast, is characterized by relatively higher and more seasonal precipitation and milder temperatures, reflecting its transitional coastal desert climate. In contrast, Hurghada, Wadi El-Gemal, and Wadi El-Galala fall within the hot desert climate zone (BWh), where extreme aridity, very low rainfall, and high temperatures dominate. Within these arid environments, Wadi El-Galala is distinguished by its higher elevation and rugged terrain, which create localized microclimatic variability and strongly influence vegetation patterns. Wadi El-Gemal, on the other hand, is notable for its pronounced habitat heterogeneity, resulting from complex geomorphological features and its status as a protected area. Collectively, this gradient from coastal to inland deserts provides a robust framework for comparing vegetation structure, species diversity, and plant adaptive strategies under varying degrees of aridity and thermal stress, justifying the selection of these floristically rich areas for the present study.

## Materials and methods

### Vegetation sampling, species classification, and conservation assessment procedures

According to^25,26^, a stratified random sampling method was applied within each area, based on physiographic features such as variations in vegetation cover and dominant species. For clarification of the sampling design, each of the 4 study areas was divided into a number of sites, and then each site included a number of sampling plots^38^. A quantitative vegetation survey was conducted across the four study areas (Fig. 1) over two successive years (2023 and 2024). Sampling sites were located away from the motorable road to minimize disturbance caused by traffic and included a diverse range of habitats: croplands, wastelands (left fallow after cultivation), orchards, sandy plains, rocky plains, salt marshes, and salinized areas. Within each site, five belt transects were taken to represent the vegetation variation, and several scattered sample plots (100m^2^) were established to comprehensively record species composition (presence/absence) and visually estimate the total plant cover. A total of 86 sampling plots were examined across the study areas: 18 plots in Hurghada, 20 plots in the Galala desert, 19 plots in Wadi El-Gemal, and 29 plots in El-Arish. Each species recorded was classified into one of four growth form categories: trees (T), shrubs (S), perennial herbs (PH), and annual herbs (AH)^35^.

Voucher specimens of all recorded species were collected, identified, and deposited at the Herbarium of Suez Canal University, Ismailia. Taxonomic identification and nomenclature followed established references^32–34^ and were cross-verified using authoritative databases, including Plants of the World Online (POWO), International Plant Names Index (IPNI), World Flora Online (WFO), and the WFO Plant List. In this study, species were classified as ‘native’ (indigenous) if their presence within a given region or ecosystem is attributed solely to natural dispersal and evolutionary processes without anthropogenic influence^35^, representing fundamental components of ecosystem composition and function. Species introduced outside their natural range through human activities, whether intentionally or unintentionally, were designated ‘non-native’ (alien, exotic, or non-indigenous), with the potential to alternative biodiversity and community structure^39,40^. Classification of each species as native (N) or non-native (NN) was performed using data from the POWO database.

The conservation status of each species was determined using the IUCN Red List^61^, the primary global reference for species conservation. Taxa were categorized according to IUCN criteria into seven categories: Critically Endangered (CR), Endangered (EN), Vulnerable (VU), Near Threatened (NT), Least Concern (LC), Data Deficient (DD), and Extinct (EX). Following^3^, endemic taxa are defined as species restricted to a specific geographic region or habitat with limited distribution, while near-endemic taxa occur predominantly within the target region but have a few records outside it. Although these taxa face significant threats to biodiversity, they are formally recognized on the Red List. In this study, recorded species were classified as endemic (E) or near-endemic (NE).

### Soil sampling and analysis

Three soil samples were collected from each plot at a depth of 0–30 cm and combined to form a composite sample^34–37^. The composite samples were air-dried, sieved through a 2 mm mesh to remove gravel and debris, and stored in paper bags for physical and chemical analyses. Soil texture was determined by hydrometer analysis to calculate the percentages of sand, silt, and clay. Soil–water extracts (1:5 ratio) were prepared to measure soil pH, chlorides (Cl^−^), bicarbonates (HCO_3_^−^), and sulfates (SO_4_^2−^). Organic matter content was also determined by loss on ignition at 600 °C for 3 h. Soil pH was measured using a pH meter (Model 206, Lutron Corporation); meanwhile, electrical conductivity (EC) was determined using a conductivity meter (Model DA-1, Lamotte Chemical). Chlorides were quantified by direct titration with 0.01N AgNO_3_ using 5% potassium chromate as an indicator^41^. Bicarbonates were also determined by titration methodology using 0.1N HCl and methyl orange as an indicator (MAFF 1986). Sulfates were measured turbidimetrically as barium sulfate. Sodium (Na) and potassium (K) concentrations were analyzed using a Drlang M 7D flame photometer, magnesium (Mg) by atomic absorption spectrophotometry (Buck Scientific Model 210 VGP), and calcium (Ca) by EDTA titration procedure.

### Data processing and statistical analysis

Classification and ordination analyses were conducted using PC-ORD version 5.0^42^ and CANOCO version 4.56; meanwhile, statistical analyses were performed with SPSS version 10.0 (SPSS, 1999). Species with frequencies below 5% were excluded prior to analysis. Two-way cluster analysis was applied to a presence/absence matrix of 86 plots and 45 species using Sørensen (Bray–Curtis) distance and flexible beta linkage with a beta value of –0.85 to group plots and species based on similarity.

Detrended Correspondence Analysis (DCA) was used to identify the main environmental gradients structuring species distributions and to quantify species turnover^43^. The first DCA axis exhibited a gradient length exceeding 7.0 standard deviation units, indicating a strong compositional turnover and supporting the application of unimodal ordination methods. Indicator Species Analysis (ISA) was subsequently performed to identify species that characterize each cluster group^44^. ISA calculates an indicator value (IV) for each species based on its relative abundance and frequency within groups. The statistical significance of IVs was evaluated using a Monte Carlo permutation test with 1000 iterations, testing the null hypothesis of no group differentiation.

To evaluate the significant differences among cluster groups, the Multi-Response Permutation Procedure (MRPP), in PC-ORD version 5.0^42^, was used. MRPP is a nonparametric multivariate test that evaluates whether predefined groups differ significantly by comparing within-group vegetation similarity to that expected by chance. Two test statistics were calculated: the T statistic, which measures between-group separation, where larger negative values (≤ –9.0) indicate stronger group differentiation; and the A statistic, a chance-corrected measure of within-group homogeneity, ranging from 0 to 1, with higher values reflecting greater homogeneity. Typically, A values are low (< 0.1) in diverse communities with many species. Statistical significance was determined through a Monte Carlo permutation test with 1000 iterations, assessing the null hypothesis of no difference among groups.

Species richness (SR) was calculated for each cluster group as the average number of species per stand. Based on the frequency (f%) of each species, the Shannon–Wiener index (H’) was used to estimate the relative species evenness according to the following equation:

$${\mathrm{H}}^{\prime} \, = \, - \sum {\text{ pi ln pi}},$$

where pi = the presence percentage (f%) of (ith) species, which is defined as pi = fi / ∑j fj; fi denotes the frequency of species i across stands and ∑j fj is the total frequency across all species within the cluster.

These diversity indices are commonly used in ecological investigations^45,46,49^ for each cluster group were measured. The software Multi-Variate Statistical Package (MVSP) version 3.13 g^47^ was used for diversity measurements. Meanwhile, ß-diversity among the 7 cluster vegetation groups (A–G) was assessed using the Chao-Jaccard index, which accounts for unseen shared species and is particularly suited for evaluating similarity between samples of different sizes with many rare species^48^. The program Estimates (Windows version 7.5, 49) was used for this analysis. A second measure of ß-diversity was derived from the length of the first DCA axis, measured in standard deviations. A length of four standard deviations indicates a complete species turnover^50^. Both α- and β-diversity are essential for understanding biogeographic patterns and the ecological processes driving them, making their integration into research crucial^51^.

Canonical Correspondence Analysis (CCA) was also performed to assess the relationships among vegetation gradients and the measured soil variables^52^. The significance of the eigenvalues for the first canonical axis was tested using a Monte Carlo permutation test with 499 permutations^53^. Prior to analysis, all soil variables were checked for normality and transformed as needed. Variables with high multicollinearity (K, Mg, Cl, HCO_3_, and SO_4_) were excluded from the CCA, resulting in eight variables used: sand, silt, clay, organic matter (OM), soil pH, electrical conductivity (EC), sodium (Na), and calcium (Ca). Significant differences among cluster groups were further evaluated using one-way Analysis of Variance (ANOVA) in SPSS version 16.0. For data visualization, RAW Graphs was used to generate Chord and Sankey diagrams, while RTutor version 0.99, integrated with the *vegan* package in R, was applied for additional ecological statistical analyses.

Plant species recorded in the studied areas were classified into life-form categories following the Raunkiaer system (1934), which is based on the position of the perennating buds in relation to the soil surface. Hemicryptophytes were defined as perennial plants whose renewal buds are located at or just below the soil surface, allowing them to survive unfavorable dry and hot periods through partial dieback of aerial shoots. Geophytes were identified as species possessing underground storage organs such as bulbs, rhizomes, corms, or tubers, with perennating buds located below the soil surface, enabling survival during prolonged drought and high temperature conditions. This classification approach is widely applied in arid and semi-arid ecosystem studies, as it reflects plant adaptive strategies to climatic stress, particularly water scarcity and temperature extremes. In hyper-arid environments such as Wadi El-Galala, Hurghada, and Wadi El-Gemal, hemicryptophytes and geophytes represent important adaptive life forms, as their growth strategies reduce water loss and allow persistence under extreme aridity and irregular rainfall.

## Results

### 1Floristic composition

**Fig. 3:**
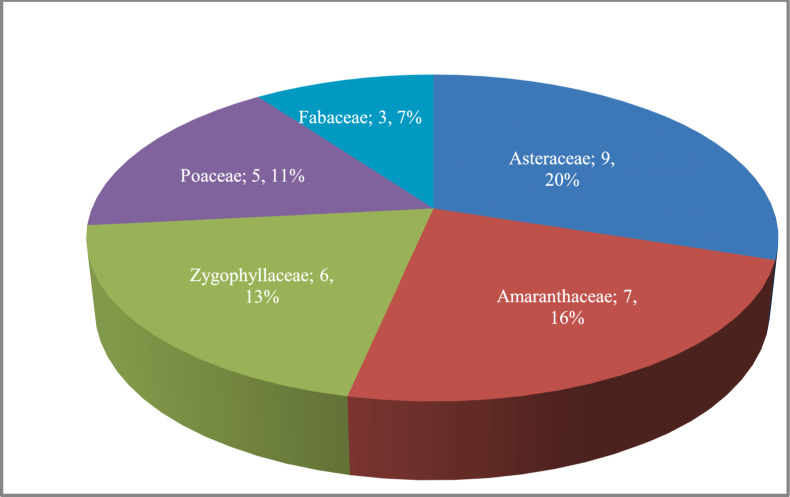
Numbers and percentages of species in the largest families. That shows *Asteraceae* as the largest family, followed by *Amaranthaceae*, *Zygophyllaceae*, *Poaceae*, and *Fabaceae.*

Forty-five species (29 perennials, 16 annuals) belonged to 16 families of vascular plants were recorded from the 86 sampled plots in the 4 studied areas. The largest families were Asteraceae (9 species; 20% of the total flora), followed by Amaranthaceae (7 species; 16%), Zygophyllaceae (6 species; 13%), Poaceae (5 species; 11%) and Fabaceae (3 species; 7%) (Fig. 3). These five families constituted 30 species or 67% of the flora, while the remaining 11 families were small sized including 1–2 species, such as Apocynaceae, Chenopodiaceae, Resedaceae, Boraginaceae, and Malvaceae. The largest genera were *Zygophyllum* (5 species), *Launaea* (3 species), *Tamarix, Bassia*, and *Astragalus* (2 species for each)^27-31^.

**Fig. 4:**
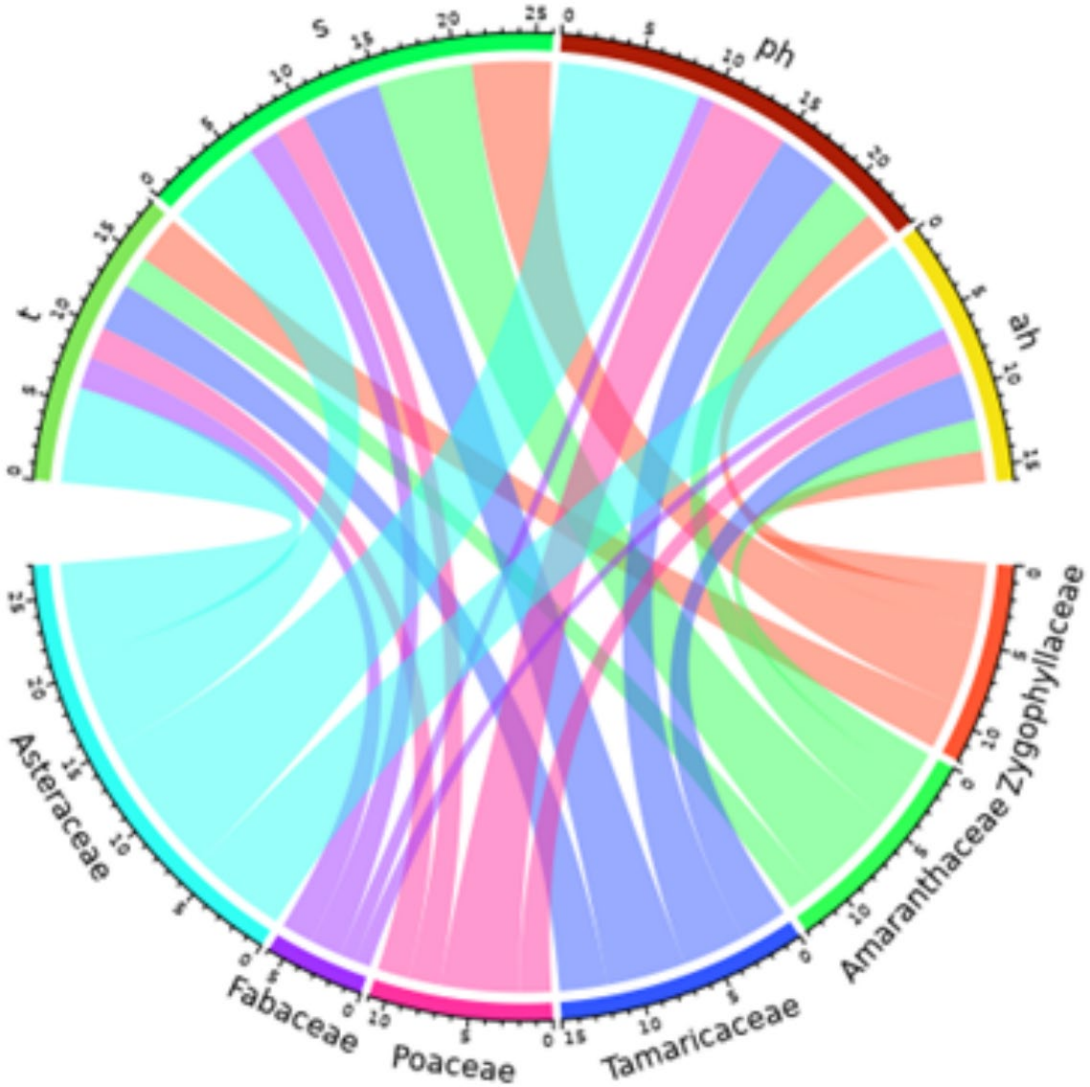
Distribution of growth forms within the major families. Growth forms: T = Trees, S = Shrubs, PH = Perennial herbs, AH = Annual herbs.

The plant growth forms varied among different species. The four species were trees (T) such as: *Tamarix nilotica, Tamarix aphylla, Vachellia tortilis,* and *Balanites aegyptiaca.* Fourteen species were shrubs (S) or subshrub (SS) as*: including Anabasis articulata, Arthrocaulon macrostachyum, Haloxylon salicornicum, Cornulaca monacantha, Zilla spinosa,* and others. There were 9 Perennial herbs (PH) such as: *Aerva javanica, Cynanchum acutum, Launaea spinosa, Echinops spinosissimus, Launaea nudicaulis* and others. In addition, 18 annual herbs (AH) were recorded, such as: *Tetraena simplex, Zygophyllum indicum, Solanum nigrum, Rumex spinosus, Hordeum murinum,* and other plants. The distribution of different growth forms within the largest families (with the highest number of species) was shown in (Fig. 4). The highest number of annual herbs (6 species) was found in Asteraceae, whereas shrubs were best represented in Amaranthaceae (4) and Zygophyllaceae (3). The 4 tree species are shared between Tamaricaceae (2), Zygophyllaceae, and Fabaceae (one for each).

The analysis of the current flora revealed the dominance of native species, with 43 species (95.5%) identified, while only two non-native species (*Erigeron bonariensis* and *Bassia indica*) were documented. Additionally, the recorded species were classified as near endemic, with no truly endemic species observed.

**Fig. 5:**
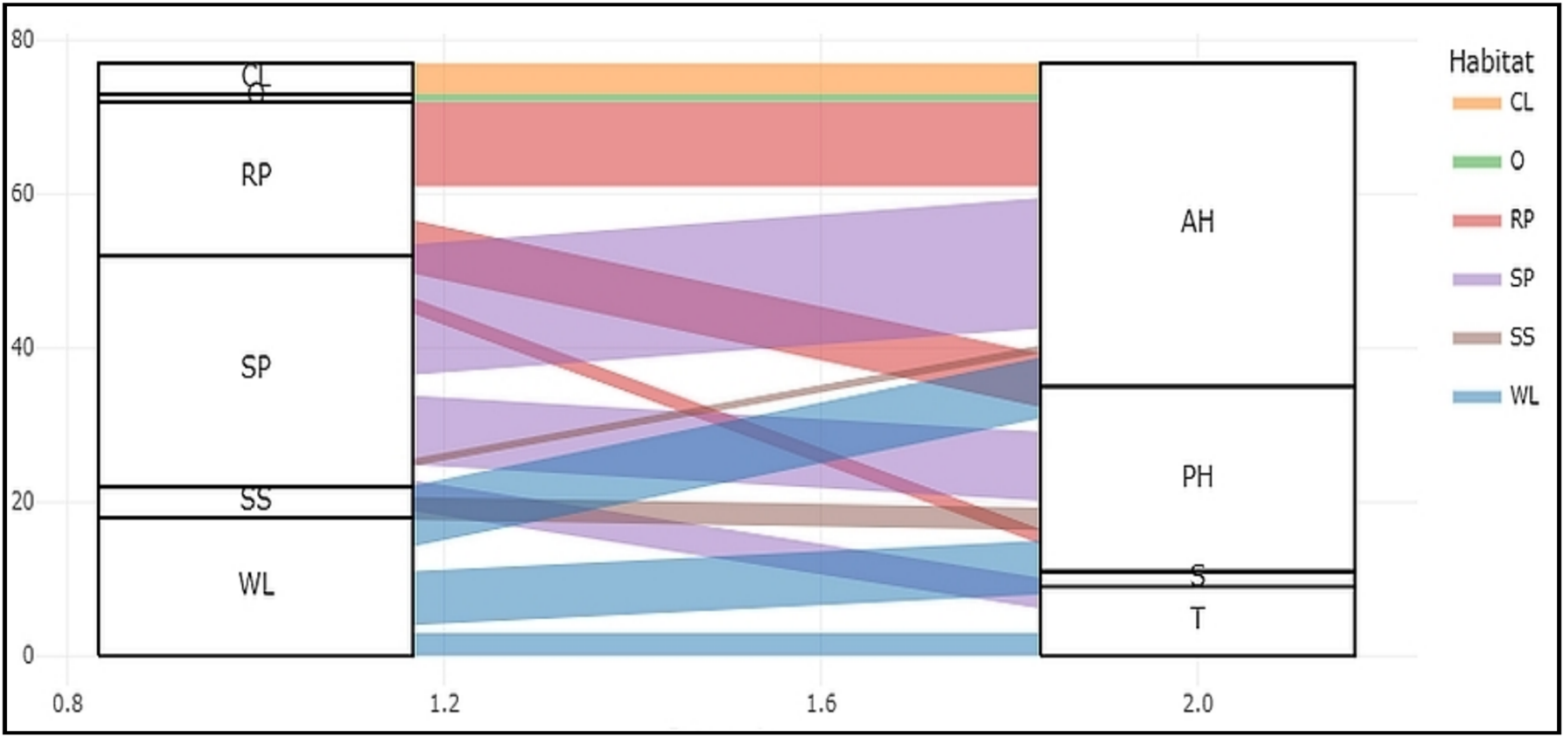
Distribution of growth forms within different recognized habitats: CL = Croplands, WL = Waste lands, O = Orchards, SP = Sandy plains, RP = Rocky plains, SS = Salinized lands. Growth forms: T = Trees, S = Shrubs, PH = Perennial herbs, AH = Annual herbs.

The relationship between different growth forms across the identified habitats is illustrated in (Fig. 5). Notably, shrubs were absent from all habitats. Annual herbs (AH) exhibited the highest abundance in sandy plains (SP) and rocky plains (RP), with 17 and 11 species recorded, respectively. A similar trend was observed for perennial herbs (PH), which were most prevalent in sandy plains and rocky plains. Conversely, cultivated lands (CL) and orchards (O) were characterized by the dominance of annual herbs. Salinized lands (SS), however, showed a predominance of perennial herbs.

The IUCN Red List^34^ categorized the conservation status of plant species based on their risk of extinction, providing crucial information for biodiversity conservation efforts. The distribution of IUCN categories within families was diverse. In Amaranthaceae *Anabasis articulata* and *Haloxylon salicornicum* were Vulnerable (VU), whereas *Arthrocaulon macrostachyum* and *Cornulaca monacantha* were classified as Least Concern (LC). Within the Asteraceae, *Launaea spinosa* and *Senecio glaucus* were categorized as Least Concern (LC), while *Pulicaria undulata* was Not Evaluated (NE), and *Reichardia tingitana* was categorized as Data Deficient (DD). In the Poaceae, *Panicum turgidum* was identified as Vulnerable (VU), while *Phragmites australis* and *Cynodon dactylon* were categorized as Near Threatened (NT).

**Fig. 6:**
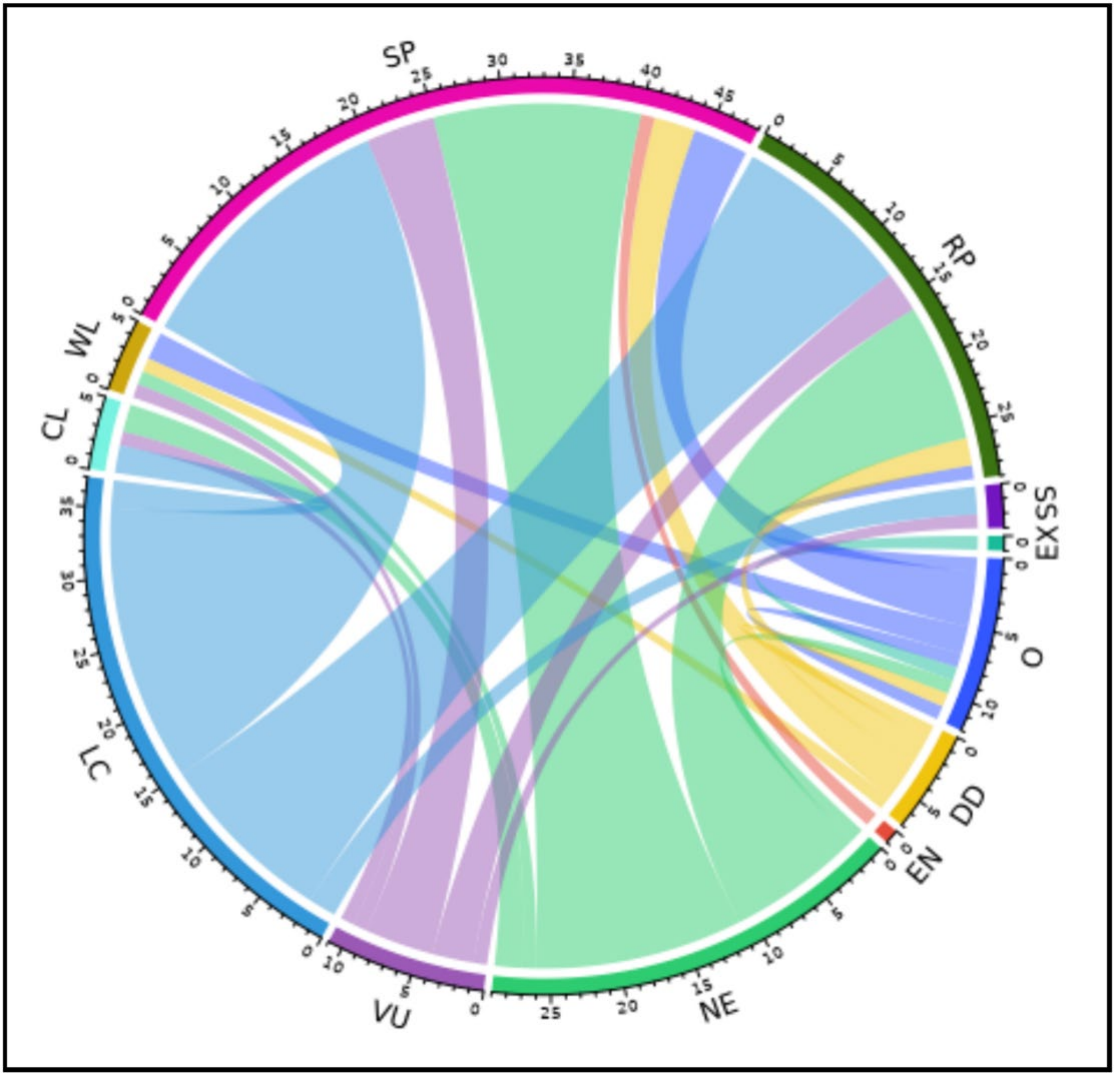
Relationship between habitats and IUCN categories. Habitats: CL = Croplands, WL = Waste lands, O = Orchards, SP = Sandy plains, RP = Rocky plains, SS = Salinized lands. IUCN categories after removal of not represented categories): EX = Extinct, EN = Endangered, VU = Vulnerable, LC = Least concern, DD = Data deficient, NE = Not evaluated.

The sandy plains showed a notable presence of species in the Least Concern (LC) and Not Evaluated (NE) conservation categories (Fig. 6), with 20 and 15 species, respectively, while the rocky plains had 13 and 10 species in these categories. Neither Critically Endangered (CR) nor Near Threatened (NT) species were found in any of the habitats.

**Fig. 7:**
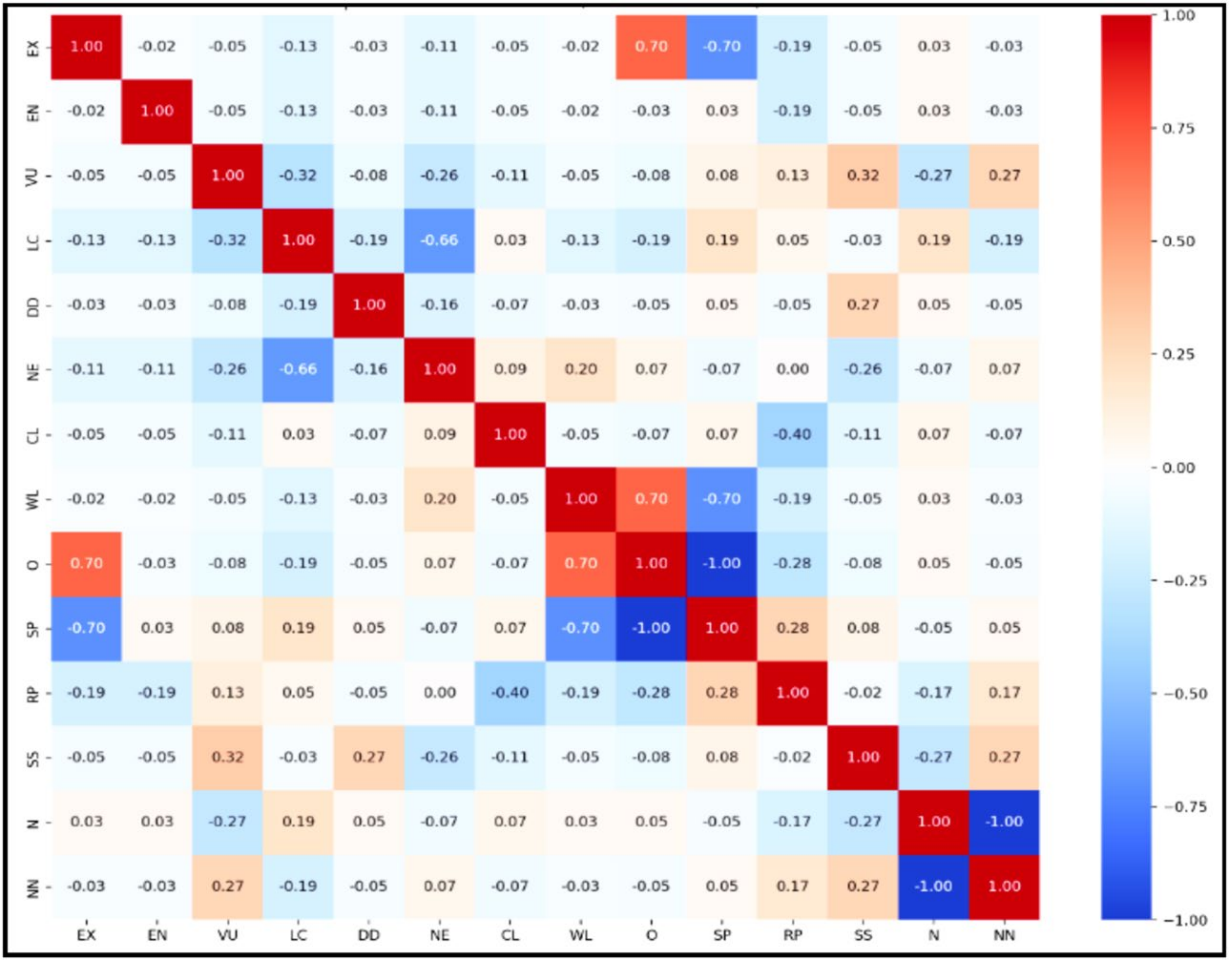
Spearman correlation matrix between habitats: CL = Croplands, WL = Waste lands, O = Orchards, SP = Sandy plains, RP = Rocky plains, SS = Salinized lands, IUCN categories after removal of not represented categories.: EX = Extinct, EN = Endangered, VU = Vulnerable, LC = Least concern, DD = Data deficient, NE = Not evaluated, and nativity: N = Native, NN = Non-native.

Figure 7 displayed Spearman correlations among habitats, categories by IUCN, and nativity, showing weak correlation coefficients for most variables. However, a strong negative correlation was observed between EX and SP (-0.70), while a strong positive correlation was found between EX and O (0.70). The correlations among IUCN conservation status categories revealed a strong negative relationship (-0.66) between Least Concern (LC) and Not Evaluated (NE). Additionally, waste lands (WL) showed significant correlations with both SP and O, with a negative correlation for the latter and a positive correlation for the former.

### Classification of the vegetation

**Fig. 8:**
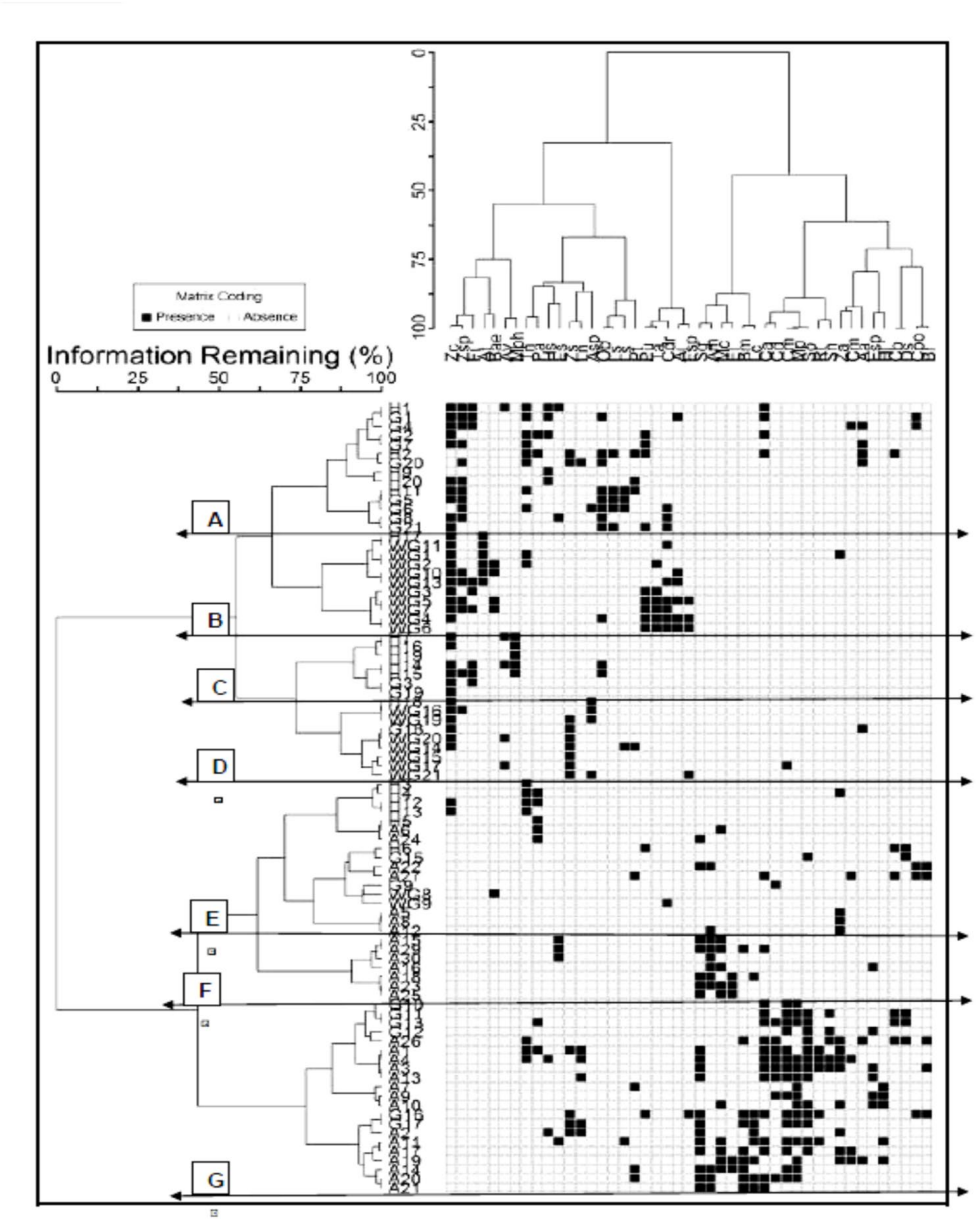
Dendrogram of the two-way cluster analysis of 86 stands and 45 species based on Sørensen (Bray–Curtis) distance measure and flexible beta resulting in 7 groups (A–G). H = Hurghada, G = Galala, WG = Wadi El-Gemal, A = El-Arish. Source: McCune, B. & Mefford, M.J. (2016). PC-ORD: Multivariate Analysis of Ecological Data. MjM Software Design, Gleneden Beach, Oregon, USA. Available at: https://mjmsystems.com/pcord.

Two-way cluster analysis was applied to a presence/absence data matrix of 86 sample plots and 45 species using Sørensen (Bray–Curtis), and a dendrogram was obtained in Fig. 8, with 7 groups (A – G) can be identified. It was found that each group can be linked to one (or more) species. The multi-response permutation procedure (MRPP) revealed that there were significant differences between these groups in the environmental matrix (chance-corrected within-group agreement *A* = 0.257; *p* < 0.0001), suggesting that these communities are distinct species assemblages. The average within-group distance ranged between 0.49 and 0.90, indicating relatively high dispersion. The *T* statistic was -31.52, indicating strong dissimilarity in plant communities (cluster groups) among the studied habitats. The pairwise comparisons revealed significant differences between the groups (Table 1). The seven groups occupied different positions of the species space, as shown by the strong chance-corrected within-group agreement (*A*) and test statistics (*T*).

**Table 1:** Multiple pairwise comparisons of the MRPP statistics of the cluster groups (A–G) of 86 sample plots in the studied areas based on Bray–Curtis’s distance.

Group compared	*T*	***A***	***P***
A vs. B	− 8.28	0.11	0.00000054**
A vs. C	− 8–10	0.14	0.00000207**
A vs. D	− 9.09	0.15	0.00000060**
A vs. E	− 9.40	0.08	0.00000011**
A vs. F	− 11.44	0.24	0.00000098**
A vs. G	− 17.87	0.18	0.00000010**
B vs. C	− 6.50	0.17	0.00005549**
B vs. D	− 8.62	0.19	0.00000516**
B vs. E	− 9.34	0.11	0.00000026**
B vs. F	− 10.02	0.31	0.00000734**
B vs. G	− 17.70	0.23	0.00000010**
C vs. D	− 6.07	0.18	0.00012369**
C vs. E	− 8.65	0.12	0.00000118**
C vs. F	− 7.88	0.35	0.00013909**
C vs. G	− 14.87	0.21	0.00000001**
D vs. E	− 9.75	0.12	0.00000014**
D vs. F	− 8.93	0.31	0.00003623**
D vs. G	− 14.96	0.19	0.00000010**
E vs. F	− 7.80	0.11	0.00000478**
E vs. G	− 12.42	0.09	0.00000011**
F vs. G	− 10–72	0.15	0.00000027**

*A* = change-corrected within group agreement, *T* = difference between the observed and expected deltas, ** = *p* < 0.01.

**Fig. 9:**
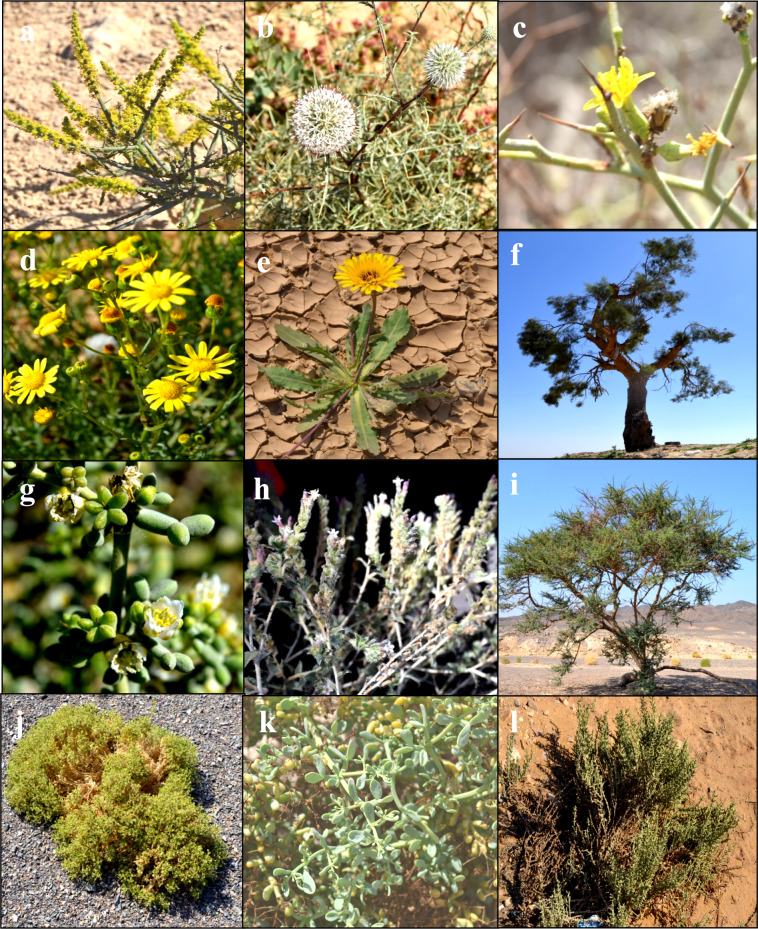
Pictures of some plant species from the 45 studied species. **a** *Ochradenus baccatus*, **b** *Echinops spinosus*, **c** *Launaea spinosa,* **d** *Senecio glaucus*, **e** *Reichardia tingitana*, **f** *Tamarix nilotica.* **g** *ygophyllum aegyptium,* **h** *Moltkiopsis ciliata*, **i** *Vachellia tortilis,* **j** *Cleome droserfolia*, **k** *Zygophyllum coccineum*, **l** *Arthrocaulon salicornicum. * *Source* Photos taken by Abdelraouf Moustafa.

**Group (A)** comprised 23 species from 14 sampled plots, predominantly occupying the sandy plains (SP) of inland desert areas in El-Galala (9 plots) and Hurghada (5 plots). This group was characterized by six indicator species: *Zilla spinosa*, *Lycium shawii*, *Ochradenus baccatus*, *Tamarix nilotica*, *Haloxylon salicornicum*, and *Anabasis articulata* (Table 2). Occasional psammophytes included *Launaea nudicaulis*, *Aerva javanica*, and *Calligonum polygonoides*. The soil in this group (Table 4) exhibited the highest concentrations of calcium (Ca), magnesium (Mg), chloride (Cl), sulfate (SO_4_), and bicarbonate (HCO_2_), along with the lowest pH values. **Group (B)** consisted of 13 species from 11 plots, with most (10 plots) located in the inland desert of Wadi El-Gemal (WG), occupying the sandy plains habitat. This group was indicated by seven species: *Tamarix aphylla*, *Aerva javanica var. javanica*, *Cleome droserifolia*, *Balanites aegyptiaca*, *Zygophyllum coccineum*, *Pulicaria undulata* subsp. *undulata*, and *Zygophyllum indicum*. Other occasional species included *Tetraena alba*, *Ochradenus baccatus*, and *Tamarix nilotica*. The soil in this group was notably fertile, with the highest contents of organic matter, fine sediments (silt and clay), and pH values. Group (C) included six species from seven plots occupying the sandy plains of the coastal desert area in Hurghada (5 plots) and the inland desert of El-Galala (2 plots). It was indicated by *Morettia philaeana* and *Zygophyllum arabicum* (Table 2). Less common species included *Zilla spinosa*, *Astragalus vogelii*, and *Ochradenus baccatus*. The soil in this group contained the highest levels of sodium (Na) and potassium (K) ions (Table 4) and had low species richness, averaging 2.7 ± 0.83 species plot^−1^. Group (D) comprised 10 species from nine plots, seven of which were in the inland desert of Wadi El-Gemal. This group was indicated by *Tetraena simplex* and *Astragalus spinosus*. Occasional psammophytes recorded included *Cornulaca monacantha*, *Panicum turgidum*, and *Anabasis articulata*. The soil in this group was characterized by the highest silt content and the lowest bicarbonate (HCO_2_) levels. Some of these plant species can be shown in (Fig. 9).

**Table 2:** Summary of indicator species analysis (ISA) showing the most significant indicator species per cluster groups (A–G).

Species	Observed indicator value (IV)	Indicator value from randomized groups		*p*
		Mean	S.D	
Group A (6 significant indicator species)				
*Zilla spinosa* (L.) Prantl	38.3	10.4	4.66	0.0006**
*Lycium shawii* Roem. and Schult	35.7	7.7	4.76	0.0004**
*Ochradenus baccatus* Delile	34.4	9.4	4.58	0.0016**
*Tamarix nilotica* (Ehrenb.) Bunge	28.9	10.7	4.59	0.0080**
*Haloxylon salicornicum* (Moq.) Bunge ex Boiss	28.2	8.3	4.79	0.0028**
*Anabasis articulata* (Forssk.) Moq	16.6	8.3	4.72	0.0442*
Group B (7 significant indicator species)				
*Tamarix aphylla* (L.) H.Karst	54.5	8.1	4.91	0.0002**
*Aerva javanica* var*. javanica*	39.3	8.1	4.92	0.0010**
*Cleome droserifolia* (Forssk.) Delile	36.3	9.2	4.64	0.0010**
*Balanites aegyptiaca* (L.) Delile	31.3	7.9	4.93	0.0042**
*Zygophyllum coccineum* L	25.3	13.5	3.87	0.0130*
*Pulicaria undulata* (L.) C.A. Mey. subsp. *Undulate*	24.4	9.4	4.56	0.0112*
*Zygophyllum indicum* (Burm.f.) Christenh. and Byng	17.2	7.8	4.71	0.0472*
Group C (2 significant indicator species)				
*Morettia philaeana* (Delile) DC	71.4	7.8	4.87	0.0002**
*Zygophyllum arabicum* (L.) Christenh. and Byng	20.1	8.9	4.72	0.0426*
Group D (2 significant indicator species)				
*Tetraena simplex* L	54.4	9.9	4.65	0.0002**
*Astragalus spinosus* (Forssk.) Muschl	38.3	7.8	4.83	0.0006**
Group E (no significant indicator species)				
Group F (5 significant indicator species)				
*Arthrocaulon macrostachyum* (Moric.) Piirainen and G.Kadereit	65.7	9.4	4.44	0.0002**
*Moltkiopsis ciliata* (Forssk.) I.M.Johnst	50.5	9.4	4.50	0.0002**
*Senecio glaucus* L	36.4	11.0	4.39	0.0012**
*Erodium laciniatum* (Cav.) Willd	35.1	7.8	4.78	0.0006**
*Echinops spinosissimus* subsp. *Spinosissimus*	29.7	8.0	4.88	0.0052**
Group G (11 significant indicator species)				
*Malva parviflora* L	71.4	10.1	4.42	0.0002**
*Sonchus oleraceus* L	56.5	9.9	4.44	0.0002**
*Cynanchum acutum* L	34.6	11.0	4.52	0.0014**
*Cynodon dactylon* (L.) Pers	33.0	8.9	4.58	0.0022**
*Bassia muricata* (L.) Asch	27.7	9.0	4.70	0.0048**
*Hordeum murinum* subsp. *leporinum* (Link) Arcang	23.8	7.7	4.62	0.0094**
*Launaea spinosa* (Forssk.) Sch. Bip. ex Kuntze	23.3	8.6	4.82	0.0162*
*Solanum nigrum* L	23.8	7.8	4.82	0.0118*
*Reichardia tingitana* (L.) Roth	23.8	7.7	4.75	0.0110*
*Tetraena alba* L.f	20.5	9.8	4.57	0.0262*
*Launaea nudicaulis* (L.) Hook.f	18.3	8.0	4.75	0.0302*

Relative indicator values (IV %) of perfect indication, and *p*-values of the most significant indicator species per group. *P-value* = Monte Carlo test of significance of the observed maximum indicator value for each species based on 4999 randomizations* = *p* < 0.05, ** = *p* < 0.001: S.D. = Standard deviation.

**Group (E)** was not characterized by any specific indicator species; however, it comprised 19 species distributed across 17 sample plots from four study areas: six from Hurghada, two from El-Galala, two from Wadi El-Gemal, and seven from El-Arish. This group included diverse habitats within coastal and inland desert ecosystems. Notable species such as *Phragmites australis*, *Tetraena alba*, and *Arthrocaulon macrostachyum* exhibited high frequency percentages (f% = 29.4%, 23.5%, and 11.8%, respectively), harboring the salinized lands of Wadi El-Gemal and El-Arish. Additionally, several weed species (*Chenopodiastrum murale*, *Sonchus oleraceus*, *Cynodon dactylon*, and *Senecio glaucus*) with f% = 11.1% were recorded in the orchards of El-Arish. **Group (F)** included nine species sampled from seven plots in El-Arish and was indicated by five species across three main habitats: *Senecio glaucus* and *Erodium laciniatum* (orchards), *Arthrocaulon macrostachyum* (salinized lands), and *Moltkiopsis ciliata* and *Echinops spinosissimus* subsp. *spinosissimus* (sandy plains). This group had the lowest diversity indices, with species richness averaging 2.1 ± 1.6 species plot^−1^ and a Shannon–Wiener index of 0.38 ± 0.62, making it the least diverse group. Its soil exhibited the highest sand content and electrical conductivity, coupled with the lowest levels of most examined soil parameters (Table 4). Occasional species in this group included *Bassia muricata*, *Launaea spinosa*, and *Cynanchum acutum*.

**Group (G)** comprised 32 species sampled from 21 plots in El-Galala (inland desert) and El-Arish (coastal desert). This group was indicated by 11 species, associated with orchard habitats in El-Arish and the sandy plains of both Galala and El-Arish (Table 2). Indicator species included several weeds (*Malva parviflora*, *Sonchus oleraceus*, *Hordeum murinum* subsp. *leporinum*, *Solanum nigrum*, *Cynodon dactylon*, *Reichardia tingitana*, and *Cynanchum acutum*), salt-tolerant species (*Tetraena alba*), and psammophytes (*Bassia muricata*, *Launaea spinosa*, and *Launaea nudicaulis*). While the soil in this group was characterized by high sand content, other soil variables showed moderate values. This group exhibited the highest diversity indices; with species richness averaging 7.9 ± 3.00 species plot ^−1^ and a Shannon–Wiener index of 1.99 ± 0.43.

The species distributions within the seven identified clusters (Groups A–G) are detailed in (Table 3). Six dominant species: *Zygophyllum coccineum*, *Zilla spinosa*, *Tamarix nilotica*, *Cynanchum acutum*, *Panicum turgidum*, and *Chenopodium murale,* were recorded in four to five groups across diverse habitats, demonstrating a wide ecological distribution. Conversely, nine occasional species displayed narrower distributions, including trees (*Lycium shawii*, *Vachellia tortilis*, and *Tamarix aphylla*), a desert shrub (*Cornulaca monacantha*), and annual herbs in Group G.

**Table 3:** Species composition of the obtained cluster groups (A–G), with their frequencies (f %).

Species	Cluster groups						
	A	B	C	D	E	F	G
Dominant species (occur 5–4 groups)							
*Zygophyllum coccineum* L	71	91	86	67	12	0	0
*Zilla spinosa* (L.) Prantl	71	36	14	11	0	0	0
*Tamarix nilotica* (Ehrenb.) Bunge	57	18	0	0	23	0	14
*Cynanchum acutum* L	28	0	0	0	6	14	62
*Panicum turgidum* Forssk	21	0	0	11	6	0	14
*Chenopodium murale* L	7	0	0	11	6	0	71
Common species (occur in 3 groups)							
*Ochradenus baccatus* Delile	57	9	28	0	0	0	0
*Launaea spinosa* (Forssk.) Sch. Bip. ex Kuntze	36	0	0	11	0	0	5
*Anabasis articulata* (Forssk.) Moq	28	0	0	11	0	0	9
*Cleome droserifolia* (Forssk.) Delile	21	54	0	0	6	0	0
*Zygophyllum indicum* (Burm.f.) Christenh. and Byng	21	27	43	0	0	0	0
*Zygophyllum simplex* L	14	0	0	78	0	0	19
*Phragmites australis* (Cav.) Trin. ex Steud	14	0	0	0	29	0	9
*Echinops spinosissimus* subsp*. Spinosissimus*	14	0	0	0	0	43	5
*Calligonum polygonoides* L	14	0	0	0	12	0	5
*Erigeron bonariensis* L	7	0	0	0	12	0	14
*Astragalus vogelii* (Webb) Bornm	7	0	28	22	0	0	0
*Zygophyllum arabicum* (L.) Christenh. and Byng	0	27	0	11	0	0	5
*Zygophyllum album* L.f	0	9	0	0	23	0	38
*Senecio glaucus* L	0	0	0	0	12	71	57
*Arthrocaulon macrostachyum* (Moric.) Piirainen and G.Kadereit	0	0	0	0	12	86	14
*Moltkiopsis ciliata* (Forssk.) I.M.Johnst	0	0	0	0	6	71	24
Less common species (occur in 2 groups)							
*Haloxylon salicornicum* (Moq.) Bunge ex Boiss	36	0	0	0	0	0	9
*Pulicaria undulata* (L.) C.A.Mey	28	45	0	0	0	0	0
*Launaea nudicaulis* (L.) Hook.f	7	0	0	0	0	0	24
*Aerva javanica* var. *javanica*	7	45	0	0	0	0	0
*Astragalus spinosus* (Forssk.) Muschl	7	0	0	44	0	0	0
*Balanites aegyptiaca* (L.) Delile	0	36	0	0	6	0	0
*Digitaria sanguinalis *(L.) Scop	0	0	0	0	12	0	14
*Bassia indica *(Wight) A.J.Scott	0	0	0	0	12	0	14
*Sonchus oleraceus* L	0	0	0	0	6	0	62
*Cynodon dactylon* (L.) Pers	0	0	0	0	6	0	38
*Erodium laciniatum* (Cav.) Willd	0	0	0	0	0	43	9
*Bassia muricata* (L.) Asch	0	0	0	0	0	14	38
*Launaea mucronata* subsp. *cassiniana (*Jaub. and Spach) N.Kilian	0	0	0	0	0	14	33
*Rumex spinosus* L	0	0	0	0	0	14	24
Occasional species (occur in one group)							
*Lycium shawii* Roem. and Schult	21	0	0	0	0	0	0
*Vachellia tortilis* (Forssk.) Galasso and Banfi	0	54	0	0	0	0	0
*Tamarix aphylla* (L.) H.Karst	0	54	0	0	0	0	0
*Morettia philaeana *(Delile) DC	0	0	71	0	0	0	0
*Malva parviflora* L	0	0	0	0	0	0	71
*Reichardia tingitana* (L.) Roth	0	0	0	0	0	0	24
*Solanum nigrum L*	0	0	0	0	0	0	24
*Hordeum murinum* subsp. *leporinum (*L ink) Arcang	0	0	0	0	0	0	24
*Cornulaca monacantha* Delile	0	0	0	0	0	0	14

An ANOVA test (Table 4) revealed highly significant differences among the examined soil variables and diversity indices across the cluster groups (A–G), except for soil sulfates (SO_4_) content.

**Table 4:**
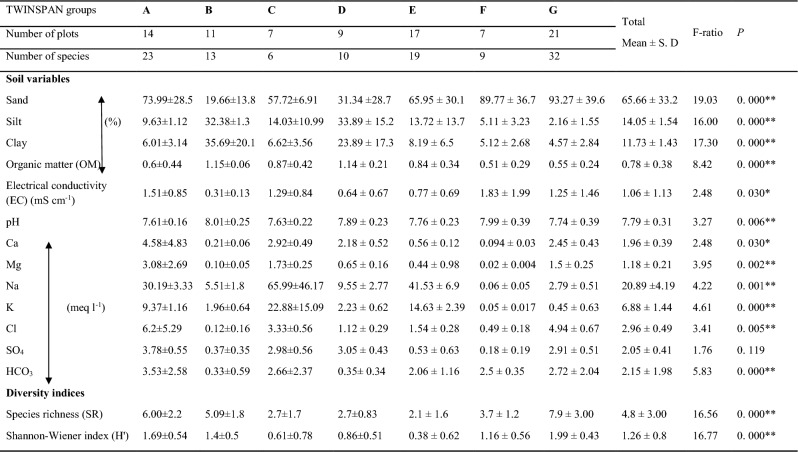
Mean values, standard deviation (± S. D) and ANOVA F values of soil variables, species richness (SR) and Shannon’s index (H’) of the 86 sampled plots representing the 7 groups obtained by cluster analysis in the study areas. ^*^ = *P* ≤ 0.05 and ^**^ = *P* ≤ 0.01.

(*T*), and cross-lagged effect (*P*20), aggregated across other conditions. Power values greater than 0.8 are indicated in bold.

### Beta (β-) diversity between cluster groups

The Chao-Jaccard similarity index (C-J) and the shared species among the 7 cluster groups (A–G; Table 5) indicated that the high species similarities (0.575) was between the groups (E) and (G), and between groups (A), (B) and (D), where the similarity index ranged between 0.441 to 0.454. In the meantime, the latter groups had higher numbers of shared species. In general, the dissimilarity between other groups was high and ranged between 0.07 (A × F) and 0.382 (F  × G).

**Table 5:** Chao-Jaccard similarity index (C-J), and the shared species among the seven cluster groups (A–G) in the 4 studied areas.

Cluster groups	Shared species	Chao-Jaccard similarity index
A × B	9	0.441
A × C	6	0.355
A × D	10	0.454
A × E	10	0.326
A × F	3	0.07
A × G	14	0.271
B × C	5	0.286
B × D	4	0.2
B × E	6	0.258
B × F	1	0.012
B × G	4	0.064
C × D	4	0.273
C × E	2	0.108
C × F	1	0.011
C × G	1	0.014
D × E	4	0.14
D × F	1	0.014
D × G	7	0.165
E × F	5	0.216
E × G	16	0.575
F × G	10	0.382

### Ordination of the vegetation

**Fig. 10:**
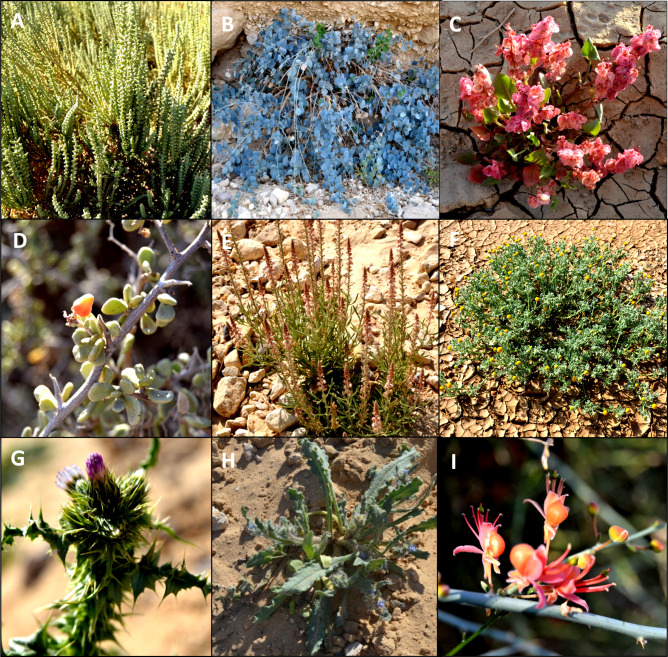
Several significant plant species were identified in the area studied; however, they were excluded from the analysis due to their frequencies being below the 5% threshold. **A** *Halocnemum strobilaceum,* **B** *Capparis spinosa,* **C** *Rumex vesicarius,* **D** *Nitraria retusa,* **E** *Oligomeris linifolia,* **F** *Cotula cinerea,* **G** *Carduus getulus,* **H** *Gastrocotyle hispida,* **I** *Capparis decidua. * *Source* photos taken by Abdelraouf Moustafa.

**Fig. 11:**
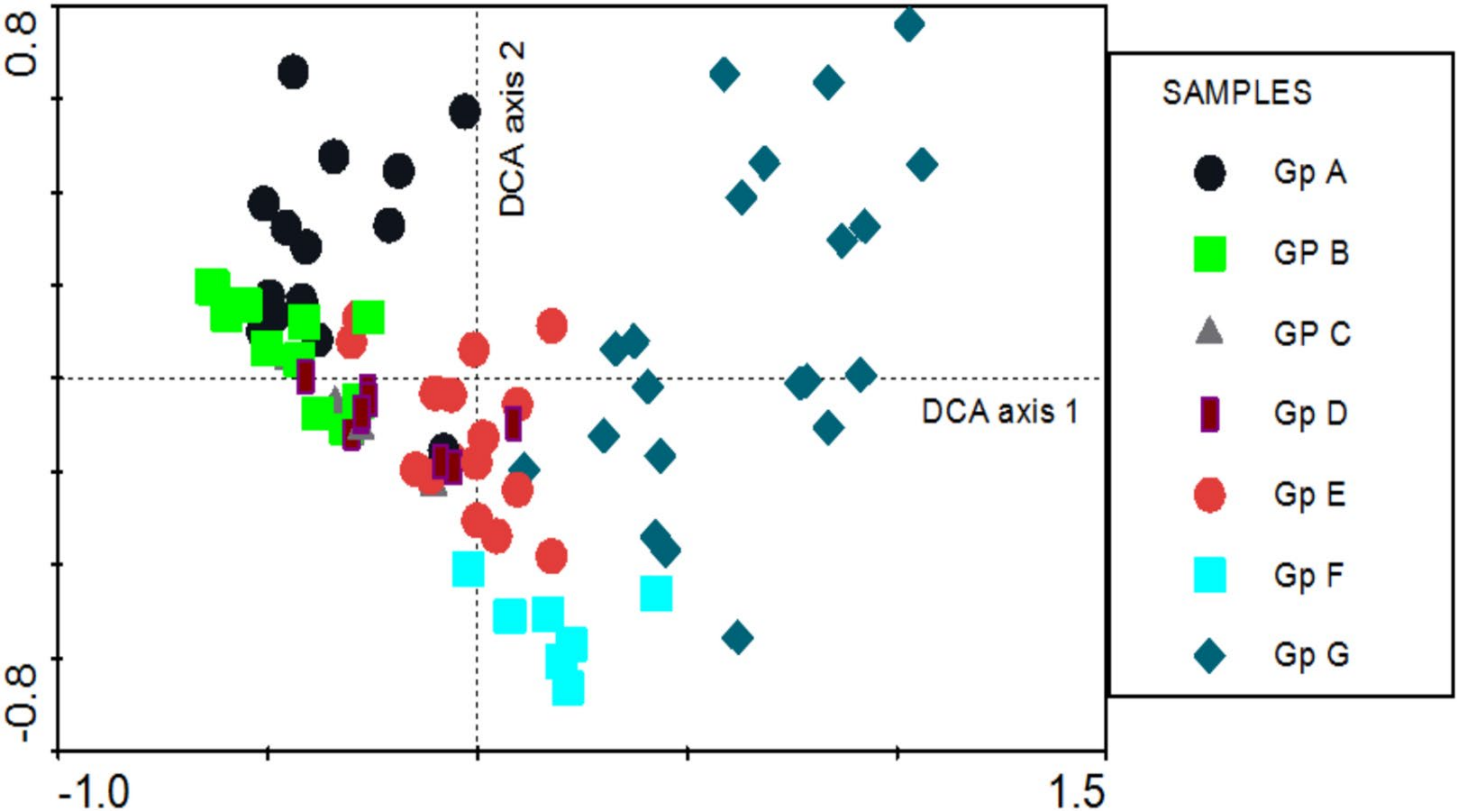
DCA scatterplot of 86 sampled plots distributed in their vegetation groups (A–G) along axis 1 (eigenvalue = 0.82) and axis 2 (eigenvalue = 0.49).

Linear response models were rejected because the gradients along the first two axes exceeded 4 standard deviation (SD) units. The gradient length for Axis 1 was greater than 7 SD, indicating a complete turnover in species composition along this gradient (Fig. 10). The four DCA axes explained 10.0%, 4.0%, 4.9%, and 4.8% of the total variation in the species data, respectively. The first DCA axis had a high eigenvalue (0.82), reflecting its significant role in capturing most of the variation in species composition across the sampled plots. The seven cluster groups (A–G) were arranged along the first DCA axis (Fig. 11). Sample plots from groups A, B, C and D were positioned at the negative end of the axis, while those from group G occupied the positive end. Groups E and F were in intermediate positions along the axis.

**Fig. 12:**
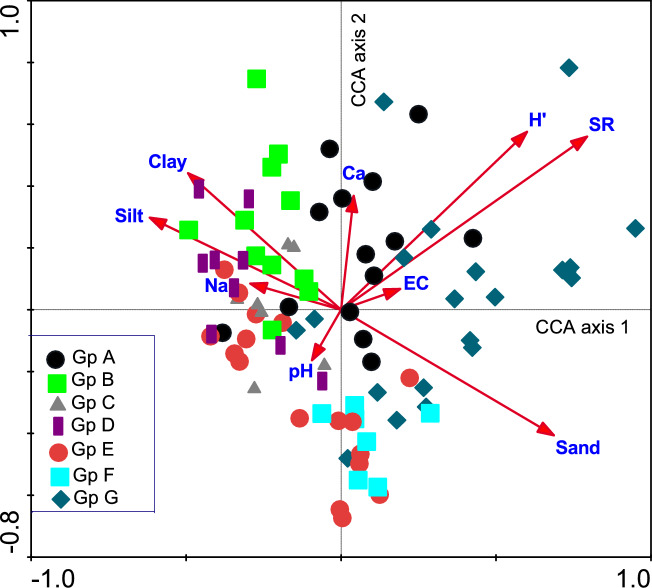
Canonical correspondence analysis (CCA) biplot of axes 1 and 2 showing the distribution of the 86 stands from the studied areas, together with their vegetation groups (A – G) and soil variables. EC = Electrical conductivity, SR = Species richness, and H’ = Shannon–Wiener diversity index.

The relationships between the results of vegetation and soil analyses using Canonical Correspondence Analysis (CCA) with the 7 vegetation groups (A–G) were shown in(Fig. 12). The biplot diagram showed a similar pattern of ordination obtained from the floristic DCA (Fig. 108), with most of the sites remaining in their respective cluster group. It can be noted that sample plots of group (A) and (G) were affected by species diversity indices (species richness and Shannon–Wiener index), Ca ions, and soil salinity (electrical conductivity). While sample plots of groups A, B, and C were correlated with soil fine sediments (silt and clay), Na ions, and pH, those of groups E and F showed no specific correlations to the examined soil variables.

Results in (Table 6) indicated the decrease of the eigenvalues of the first two CCA axes (0.552 and 0.296, respectively), suggesting a well-structured data set. The species-environment correlations were high for the four axes, explaining 67.5% of the cumulative variance. These results suggested an association between vegetation and the measured soil variables presented in the biplot.

**Table 6:**
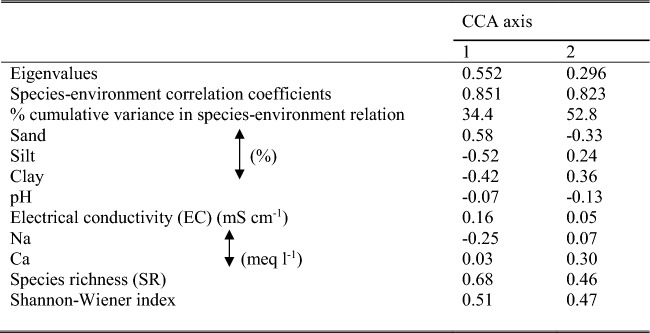
Results of inter-set correlations of the soil variables along the first two axes of CCA, together with eigenvalues and species-environment correlation coefficients.

(*T*), and cross-lagged effect (*P*20), aggregated across other conditions. Power values greater than 0.8 are indicated in bold.

The inter-set correlations resulting from CCA of the examined soil variables are shown in (Table 6). Axis 1 was positively correlated with species richness, while negatively correlated with silt. This axis 1 can be interpreted as species richness-silt gradient. Axis 2 was positively correlated with the Shannon–Wiener index and negatively correlated with sand. This axis 2 can be interpreted as the Shannon Wiener index- sand gradient. A test for significance with an unrestricted Monte Carlo permutation test found the F-ratio (10.87) for the eigenvalue of CCA axis 1 and the trace statistics to be significant (*p* = 0.002), indicating that the observed patterns did not arise by chance.

## Discussion

The analysis of species composition across different cluster groups plays a crucial role in understanding the ecological dynamics of the study area and has significant implications for conservation and management efforts. By examining the distribution and ecological requirements of indicator species, this analysis aids in the development of more effective strategies for the conservation and restoration of biodiversity within these ecosystems. Investigating the community structure of plant species through the species composition of each cluster allows for inferences about the broader structure of plant communities. For example, clusters with a higher frequency of shrubs may suggest the presence of distinct habitat types, in contrast to those dominated by grasses^54^. The spatial distribution of species across clusters can also reveal environmental gradients or factors influencing plant community composition. For instance, the consistent association of a particular species with high salinity levels could serve as a reliable indicator of saline conditions. Furthermore, the co-occurrence patterns of species within clusters provide valuable insights into ecological relationships, including competition, facilitation, or mutualism. From a conservation perspective, understanding the composition of species of various habitats enables the targeting of efforts to protect and manage specific plant communities, enhancing the effectiveness of conservation interventions. This approach, by utilizing statistical data and generating species lists for specific locations, offers a robust framework for analyzing plant communities and their conservation needs^54^.

Numerous studies support the notion that human activities, such as tourism and urbanization, can significantly affect the dynamics of plant populations in coastal dunes. Mohamed et al.^10^ examined the impact of coastal development on dune vegetation and found that urbanization leads to habitat fragmentation, loss of biodiversity, and the proliferation of invasive species, all of which result in a marked decline in plant populations, particularly among endemic and threatened species. These findings underscore the critical need for long-term land-use planning and conservation measures to safeguard these ecologically rich ecosystems. Moreover, they highlight the importance of conducting continuous inventories of plant species and monitoring their populations to inform effective conservation strategies^10^.

The findings of this study underscore the importance of preserving Egypt’s diverse habitats, particularly coastal and desert ecosystems, which are under increasing pressure from anthropogenic activities such as agriculture, urbanization, and climate change^55^. Effective conservation strategies should focus on protecting native species and their habitats while managing invasive species and mitigating the effects of land use changes. Furthermore, ongoing monitoring of these ecosystems will be crucial to understanding the long-term impacts of climate change and human activities on plant biodiversity in Egypt.

Species richness and diversity indices indicated moderate to high biodiversity across the sampled habitats, with a Shannon–Wiener index ranging from 0.38 to 1.99. While diversity was relatively high in some areas, it is notable that several plots exhibited low species richness, particularly in disturbed habitats like cultivated lands and orchards. This finding concurs with the work of^56^, who noted that agricultural activities in Egypt contribute to habitat degradation, reducing plant diversity by altering soil conditions and increasing competition from invasive species. It’s an important indicator that research on biodiversity and soil quality indicators is necessary to more precisely evaluate the environmental degradation resulting from the transformation of deserts into agricultural zones, considering the richness of species in these areas.

Conservation status assessments showed that 95.5% of the recorded species were native, with two non-native species identified. According to (IUCN, 2024), the Amaranthaceae, species such as *Anabasis articulata* and *Haloxylon salicornicum* are categorized as Vulnerable (VU), indicating that they are at a high risk of becoming endangered unless conservation measures are implemented. Conversely, species like *Arthrocaulon macrostachyum* and *Cornulaca monacantha* are classified as Least Concern (LC), suggesting that they are currently stable with no immediate threat of extinction. Within Asteraceae, *Launaea spinosa* and *Senecio glaucus* are similarly classified as Least Concern (LC), while species such as *Pulicaria undulata* are not evaluated, indicating insufficient data to assess their status. Other species like *Reichardia tingitana* fall into the Data Deficient (DD) category, reflecting a need for more information before a reliable conservation status can be assigned. In the Poaceae family, *Panicum turgidum* is identified as Vulnerable (VU), underlining its susceptibility to environmental pressures, while *Phragmites australis* and *Cynodon dactylon* are categorized as Near Threatened (NT), highlighting their potential vulnerability without immediate conservation actions. A few species across other families, such as *Vachellia tortilis* (Fabaceae) and *Zygophyllum indicum* (Zygophyllaceae), are classified as Near Threatened (NT), which signals an increasing risk of extinction. On the other hand, species like *Zygophyllum coccineum* and *Tamarix nilotica* fall under Data Deficient (DD) or Not Evaluated (NE), showing the need for further ecological assessments to determine their conservation needs.

Zahran and Willis^57^ characterize the El Galala mountain range and its accompanying wadies as a unique habitat for several plant species suited to harsh desert conditions, exhibiting traits such as succulent leaves, deep roots, and drought-resistant mechanisms. They play a crucial role in the region’s environment by offering sustenance and habitat for wildlife, aiding in soil erosion control, and preserving overall ecological equilibrium. Consistent with our findings, they identified several desert shrubs flourishing in El Galala, including *Zygophyllum coccineum*, *Tamarix nilotica*, and *Zilla spinosa*^57^ (Zahran and Willis^57^.

The different ecosystems and the results in which species were found after applying data analysis for plant species, we find that with the agreement to our results in plant species,^24^ described that about 140 plant species from 44 families have been reported in the Wadi El-Gemal National Park, which was established to protect an extraordinary terrestrial and marine ecosystem that is extremely rich in terms of landscape, botanical, and wildlife diversity, as well as numerous archaeological sites of great significance. The specific plant species found in Wadi El-Gemal can vary depending on the location within the park, elevation, and proximity to water sources. The park’s diverse habitats, ranging from coastal areas to inland wadis, support a wide range of plant life. The prominent plant species found within this unique ecosystem include *Tamarix nilotica*, *Zygophyllum coccineum*, etc.…

Zahran et al.^9^ studied that Egypt’s coastal and inland deserts cover more than 96% of the country’s total area; these deserts are distinguished by the presence of a constant structure of halophytic and xerophytic plant types. Egyptian desert soils in general have low organic matter, with a slightly acidic to alkaline reaction at the surface, calcium carbonate accumulation in the top horizon (1.5 m, 5ft), coarse to medium texture, and low biological activity^58^. The high salinity and low pH observed in sandy plains and rocky plains likely reflect the harsh conditions that dominate these ecosystems, affecting the distribution of plant species, particularly halophytes and psammophytes. For example, *Tamarix nilotica* and *Haloxylon salicornicum*, both found in this study, are known for their tolerance to saline conditions. Despite a shortage of precipitation, the natural vegetation in Egypt’s deserts is varied. Most of the Western Desert is completely arid of plant life, but where there is water, the regular desert vegetation of perennials and grasses can be seen; and the coastal strip has a diverse plant life in the spring. Although the Eastern Desert found in Wadi El-Gemal and El Galala receives little rainfall, it supports different vegetation that includes tamarisk, *acacia*, and *markh*, as well as a wide range of thorny bushes, small succulents, and scented plants. The spaces in between these perennial plants are usually vegetated with the short-lived ephemeral, annual and biennial (therophytes) herbs that appear only during the rainy years. The development of these plants is most noticeable in the wadis of the Red Sea Hills, Sinai, and the ʿIlbah (Elba) Mountains to the southeast^9^.

On the other hand, it has been confirmed by^19^ that the area of El-Arish is covered with several wild plant species, the most prevalent of which are Athel (*Tamarix* sp.) and acacias (*Acacia* sp.). Date palms can be found in various locales. Weeds commonly found in the area include Nigeel (*Pancium* sp.) and Hagana (*Phragmites australis*). The land is primarily planted with date palm, olive, citrus, guava, and grape trees. The irrigation system is based on groundwater wells, but Nile water reaches the cultivated plains via the freshly dug Canal of El-Salam. According to^59^, which made a study on the floristic patterns of the sand-dune ecosystem across the Mediterranean coastline of Egypt and found four groups that include *Pancratium arabicum,* inhabiting the stabilized dunes and dominated by *Echinops spinosissimus*. The Mediterranean ecosystem in general is dominated by evergreen shrubs and small trees. Calcium carbonate is an important indicator of Mediterranean endemics’ distribution in Egypt. The vegetation composition of Mediterranean endemics in Egypt was classified by (TWINSPAN) classification into five vegetation groups (VGs): I: *Cyperus capitatus*, II: *Echium angustifolium subsp. sericeum*, III: *Asparagus stipularis*, IV: *Zygophyllum album,* and V: *Fumaria judaica subsp. Judaica*^59^.

In connection with our studies on plant species found in the red seacoast (in Hurghada), there is a comparable study on a nearby region located in Wadi Esli and Wadi ElMallaha close to Hurghada by^60^. The research area’s floristic richness included 23 species across 19 genera and 14 families. Overall, the major families were Zygophyllaceae, Fabaceae, Tamaricaceae, and Asteraceae. A single species represented nine families. The genus with a larger number of species was Tamarix sp. The plants they found include: *Zygophyllum coccineum* group, *Aerva javanica* (Burm.F.) Juss. ex. Schult., *Pulicaria undulata* (L.) C. A. Mey, *Zilla spinosa* Forssk, *Cleome dreserifolia* (Forssk.) Delile., *Phragmites australis* (Cav.) Trin. ex Steud, *Tamarix nilotica* L., etc.

## Conclusion

This study provides a comprehensive assessment of the biodiversity of plant species across various ecosystems in Egypt, with particular emphasis on coastal and inland desert regions. The analysis of species composition and diversity metrics across seven distinct vegetation clusters revealed notable variations in plant diversity between the surveyed habitats. These findings underscore the importance of consistent monitoring of plant species diversity to track temporal changes and guide the development of adaptive and context-specific conservation strategies. A detailed examination of plant species composition offers essential insights into ecosystem health and enhances our understanding of the ecological dynamics at play. The results highlight the significant impact of human activities, particularly in coastal areas, which pose a threat to the stability and resilience of these ecosystems. Previous studies in Egypt have documented the remarkable diversity of plant species in both coastal and inland desert regions, emphasizing the need to understand species distribution patterns and ecological requirements for effective conservation and restoration efforts. Using multivariate analysis tools such as Sørensen distance and flexible beta diversity, the study examined the relationships among 45 plant species from 16 families, with Asteraceae being the most dominant family. The analysis identified distinct clusters within the plant community, each with its own unique species composition, further highlighting the complex ecological structure of these ecosystems. These findings contribute to the growing body of knowledge on Egypt’s flora and reinforce the importance of targeted conservation interventions to preserve the nation’s diverse plant life.

## Data Availability

Data sets generated during the current study are available from the corresponding author on reasonable request. My data will be available on any request in my lab at Suez canal university, Ismailia, Egypt.
